# Detection of attractors of large Boolean networks via exhaustive enumeration of appropriate subspaces of the state space

**DOI:** 10.1186/1471-2105-14-361

**Published:** 2013-12-13

**Authors:** Nikolaos Berntenis, Martin Ebeling

**Affiliations:** 1 Non-Clinical-Safety, F. Hoffmann – La Roche AG, Grenzacherstrasse 124, 4070, Basel, Switzerland

**Keywords:** Boolean network, Attractor, Fixed state, Cycle, Regulatory network, State space

## Abstract

**Background:**

Boolean models are increasingly used to study biological signaling networks. In a Boolean network, nodes represent biological entities such as genes, proteins or protein complexes, and edges indicate activating or inhibiting influences of one node towards another. Depending on the input of activators or inhibitors, Boolean networks categorize nodes as either active or inactive. The formalism is appealing because for many biological relationships, we lack quantitative information about binding constants or kinetic parameters and can only rely on a qualitative description of the type “A activates (or inhibits) B”. A central aim of Boolean network analysis is the determination of attractors (steady states and/or cycles). This problem is known to be computationally complex, its most important parameter being the number of network nodes. Various algorithms tackle it with considerable success. In this paper we present an algorithm, which extends the size of analyzable networks thanks to simple and intuitive arguments.

**Results:**

We present *lnet*, a software package which, in fully asynchronous updating mode and without any network reduction, detects the fixed states of Boolean networks with up to 150 nodes and a good part of any present cycles for networks with up to half the above number of nodes. The algorithm goes through a complete enumeration of the states of appropriately selected subspaces of the entire network state space. The size of these relevant subspaces is small compared to the full network state space, allowing the analysis of large networks. The subspaces scanned for the analyses of cycles are larger, reducing the size of accessible networks. Importantly, inherent in cycle detection is a classification scheme based on the number of non-frozen nodes of the cycle member states, with cycles characterized by fewer non-frozen nodes being easier to detect. It is further argued that these detectable cycles are also the biologically more important ones. Furthermore, *lnet* also provides standard Boolean analysis features such as node loop detection.

**Conclusions:**

*lnet* is a software package that facilitates the analysis of large Boolean networks. Its intuitive approach helps to better understand the network in question.

## Background

The use of Boolean models I n the study of biological networks was proposed and worked out already in the 1970s [1-5]. A Boolean network model is characterized by the topology of a biological interaction network and a set of qualitative parameters termed “logical functions” by Thomas and D’Ari [6]. Logical functions determine the activation state, or value, of any node in a network as a function of its activating and/or inhibiting inputs. In general, for a target node with k different input nodes, each of which can again be either “active” or “inactive”, the logical functions assign the resulting values of the target node for each of the 2^k^ possible input patterns.

Boolean networks are best suited to analyze and describe steady states of systems (which are independent of kinetic parameters). As demonstrated by Thomas, non-trivial steady states are determined by the presence of negative or positive feedback loops, with the former characterizing homeostatic or oscillatory processes, and the latter leading to switch-like or differentiation behaviour.

The analysis of Boolean networks comprised of more than a few nodes is feasible thanks to tools developed by various groups. Garg et al. [7] introduced the concept of binary decision diagrams and developed *SQUAD*[8]. *GINsim*, a tool implementing Thomas’ program was provided by Gonzalez et al. [9]. Himkelman et al. [10] used algebraic methods to develop *ADAM*, Helikar et al. [11] developed the simulation platform *ChemicalChains* and Müssel et al. developed *BoolNet*[12].

Algorithms were also developed that simplify network architectures without affecting the steady state properties. They eliminate iteratively single nodes that: do not regulate their own function [13,14] or: have one incoming and one outgoing edge (simple *mediator* nodes) or have the same value in all attractors [15,16]. Both approaches preserve the fixed point structure of the network. The latter preserves also the cycle attractors, while the former may, in certain cases, introduce spurious ones.

For a binary Boolean network with n nodes, there are *2*^n^ possible activation patterns that form the state space: every state is represented by an n-dimensional binary vector. In the case of multi-valued Boolean networks, where some of the nodes can have more than 2 values, the number of activation patterns grows even faster. We refer to this number as the “state complexity” of a network.

**Figure 1:**
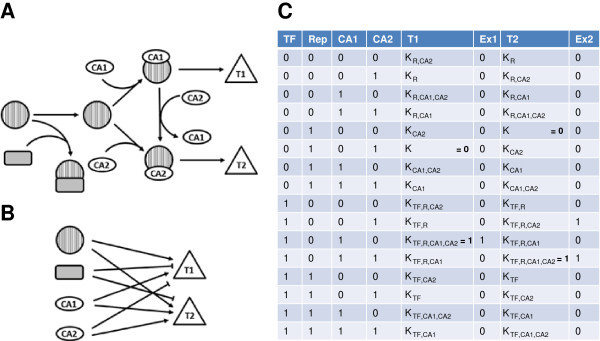
**State complexity and parameter complexity. A: **Schematic representation of a hypothetical regulatory biological network. A transcription factor TF (striped circle) interacts with either one of two competing co-activators (CA1, CA2) to drive expression of one of two target genes (triangles T1, T2). Presence of an inhibitor (grey, rounded rectangle) blocks TF. **B:** In the absence of detailed knowledge of the underlying interactions, the network from A can be simplified into this diagram, with the state complexity translated into parameter complexity as shown in **C**. **C:** For the 2^4^ possible activation patterns of the 4 input nodes in **B**, logical functions for the two targets T1 and T2 are specified. The only knowledge is about the activating or inhibiting influence of the input nodes on the targets. For the logical functions, we use the convention introduced by Thomas et al. [6], i.e. parameter K_TF,R,CA2_ stands for the value (either 0 or 1) to which the target node tends under the positive influence (i.e., presence of activators, absence of inhibitors) of the input nodes mentioned as subscripts. For *lnet*, only two of the logical parameters for every node are pre-defined (values assigned in bold). Two additional columns, Ex1 and Ex2, specify the concrete values of all parameters for the system as depicted in **A**.

For every node in a network, the logical parameters determine how it reacts to the input it receives from other nodes, more specifically, to which value (0 or 1) a node will tend under any given input pattern, also referred to as the “image” of the node for this input pattern. In general, for a node with k inputs (activating or inhibiting), there are 2^k^ possible input patterns and corresponding logical parameters. We refer to this as “parameter complexity” of a network. For the choice of logical parameters, there are some obvious constraints, e.g., adding an activating input to an already active node should not lead to its inactivation. For simplicity reasons, Boolean network analyses often adopt the convention that a node under the influence of at least one inhibitor always tends towards being inactive irrespective of the presence of any activators; in the absence of any inhibiting influences, a single activator will be sufficient to activate it. This effectively eliminates the parameter complexity of the problem. Here, we deviate from this convention, motivated by biological examples where, for example, both a transcription factor and a co-activator are required for a certain function, or where co-activators and co-repressors compete for a target transcription factor (Figure 1). The only assumption we make here is that under “optimal” conditions, i.e., presence of all activators and absence of all inhibitors, a node must be activated, and likewise, in the presence of all inhibitors and absence of any activators, it must be inactivated. These minimum assumptions ensure that every node must in principle be able to switch between its two values. We note that state and parameter complexity are related and it is often possible to reduce parameter complexity by adding symbolic nodes to the network that represent complexes or intermediate steps along a process; however, this comes at the expense of higher state complexity.

For any given network and network state, it is not obvious if all nodes satisfy the logical functions, i.e., have values in agreement with their inputs; in fact, it is not even obvious if such a state exists. Node values can be updated by switching them in agreement with the input pattern. Whenever stated that in a given state a node satisfies the equations, it is meant that the node will retain its value should we choose to update it (i.e., the value is identical to the image for the current input pattern). For simplicity, we refer to such a node as a “content” node. A fixed state of the network is, by definition, a state in which all the node equations are satisfied, i.e. all the nodes are content. In contrast, a node whose current value is in disagreement with its current input, and which will be modified when chosen for an update, is called a “discontent” node.

Boolean networks have often been studied using “synchronous” update strategies, where the values of all discontent nodes are switched simultaneously. (Note that this switching can lead to the generation of new discontent nodes.) While this approach is technically convenient, it does not properly reflect the characteristics of biological networks, and it may even introduce artifacts in their behavior. “Asynchronous” updates proceed with one node at a time, often selecting them in random order. This method, referred to as “general asynchronous” in [15], is the one we choose.

In the following, we are referring to random asynchronous updating and to binary Boolean networks (although the algorithm is also valid for multiple discrete-valued ones). Furthermore, all results were generated from testing on networks having: (i) values of the ratio of edges to nodes between 2 to 3 and (ii) all their nodes with non-zero in- and out-degrees and with at least one of them larger than one (i.e., without simple mediator nodes).

## Implementation

The software application has been written in ANSI C and it is single-threaded. The only hardware requirements concern available random access memory. The network topology can be loaded using the straightforward format also used in [7]. In the input text file each line corresponds to either an activation A → B or to an inhibition C − | D.

The source code is freely available from the authors.

## Results

### Fixed states detection

**Figure 2:**
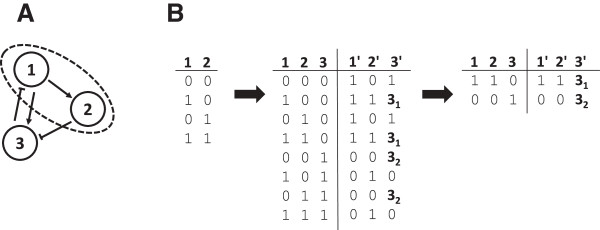
**For this simplified account of the algorithm described in the text, we analyze a 3-node network (A).** We start with the full listing of all value combinations for nodes 1 and 2 in a 2 x 22 matrix, then add node 3 as described to obtain a (2 + 1) x 22+1 matrix **(B)**. We start with the full listing of all value combinations for nodes 1 and 2 in a 2 x 2^2^ matrix, then add node 3 as described to obtain a (2 + 1) x 2^2+1^ matrix. For this, we also specify the images 1′, 2′, 3′ to which the three nodes tend for any input combination, using logical parameters. In this simple example, only node 3 receives multiple inputs and thus has non-trivial logical functions. Removing the states which cannot match their image state for any valid choice of the logical functions, we obtain two candidates for fixed states. Comparing the states to their image states, we realize that state (1, 1, 0) is a fixed state if **3**_**1**_ equals 0 (i.e., node 3 is not activated by the presence of its activator 1 alone), whereas state (0, 0, 1) is fixed if **3**_**2**_ equals 1 (i.e., node 3 is already activated by the absence of its inhibitor 2 alone). Setting both **3**_**1**_ = 0 and **3**_**2**_ = 1 renders both states fixed. Experimental data in agreement with state (1, 1, 0) but not with (0, 0, 1) thus help in estimating the logical functions.

Crucial for the development of the algorithm is the realization that the discrete nature of Boolean networks allows us to restrict the search to selected subspaces of the state space in which the fixed states (if any exist) reside. These subspaces turn out to be orders of magnitude smaller than the actual state space rendering possible an exact enumeration. This is achieved via a two-step process (Figure 2).

Assume a Boolean network of n nodes. For k of them (typically, k around 10), generate the 2^k^ × k matrix representing all their 2^k^ possible states. Then for each one of the remaining n-k nodes go through the following iterative process:

Extend the matrix by first duplicating it and then adding to it an extra column with the values of an appropriately chosen (k + 1)^st^ node (0 and 1 for each pair of identical rows). The outcome is a 2^k+1^ × (k + 1) matrix. The choice of the new node is crucial: All its incoming nodes must be present in the initial k-member group (see remark below). Then, for each row, we check whether the value of the added node satisfies its node equation. If it violates it then we remove the row, for it cannot lead to a fixed state. By doing so, all its 2^n-(k+1)^ downstream successors are effectively removed once and for all. The same procedure is followed for any of the k initially selected nodes, if all its incoming nodes are in the matrix.

Then a (k + 2)^nd^ node is added, further extending the matrix, its equation is tested against all rows, and the process is repeated until all the nodes have been taken into account. At each iteration step, if no remaining node is found having all its incoming nodes already present in the matrix, the one with the maximal out-degree is chosen. The algorithm is simply trying to increase the probability that at each step at least one node having all its incoming nodes already present will be available. The choice of the first k nodes is based on this reasoning too: the ones with the highest out-degree values are selected.

The successive removal of equation-violating states (i.e., states containing at least one discontent node) leads to a dramatic decrease of the number of states examined. It can simplify the problem by many orders of magnitude, depending on network size and complexity. In a typical network the number of examined states reaches its maximum value at about or just after the time half of the nodes have been included in the matrix. Then it decreases rapidly as many of the node equations are taken into account during the evaluation. When the full set of nodes has been processed, only the fixed states remain in the final matrix.

We note that the approach described here simultaneously copes with both state and parameter complexity. When testing if the actual value of a node is compatible with the node equations, we accept any states for which this condition holds for at least one choice of the logical parameters. Thus, the algorithm does not only yield fixed states for a concrete combination of logical parameters, but for all possible ones. The conditions on the logical parameters needed to realize a fixed point can then be read from the final matrix (Figure 2B). This aspect is of key importance for reverse-engineering problems.

The generalization to multiple-valued case is straightforward: At each iterative step, instead of just duplicating the rows, the algorithm adds m-1 replicates of the previous matrix when adding a node with m levels, with the corresponding level (from *1* to *m-1*) appended. Then it proceeds as previously described, namely by removing all equation-violating states.

### Cycle detection

#### ***Preliminaries***

The system equations direct each state to flow to neighboring states and (in asynchronous mode) neighboring states differ by the value of at most a single node. The changing node is set to a value that satisfies the system equations, which increases the number of content nodes by one; however, this change will typically have effects on other nodes, and other discontent nodes can result. If the state space contains basins of attraction then the system will eventually flow towards them. Basins of attraction signal the existence of *fixed states* or of *stable cycles*, i.e., sets of states out of which the system cannot escape once it reaches them. Once a system enters a basin of attraction then the equations drive it deeper into it, gradually increasing the number of content nodes. However, for any basin of attraction, it is not trivial to find out if it can be reached from any given current state.

**Figure 3:**
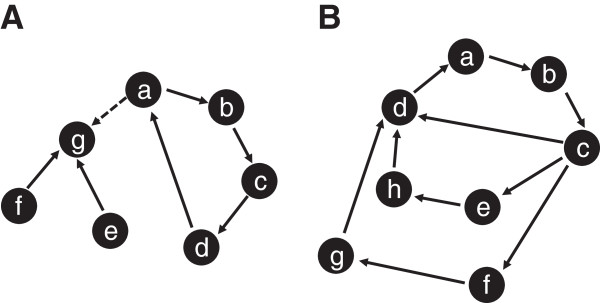
**Analysis of simple and complex cycles.** Filled circles represent states of the system, not to individual nodes. In asynchronous update mode, two neighboring states differ by the value of exactly one node. Thus, the shortest possible cycle (0, 0) → (0, 1) → (1, 1) → (1, 0) → (0, 0) → …, composed of two changing nodes, has length 4 in state space. **A:** The cycle “abcd” is fixed only in the absence of the dashed transition a → g. In the presence of this transition, the cycle leaks into state g, and the system has only a single fixed point, g. **B:** In the cycle “abcd”, here state c is 3-discontent and, hence, can transition to three different successor states d, e, and f. For the cycle to be fixed, as shown here, all the successor states of d, e, and f have to return to the cycle. As a consequence, complex fixed cycles are expected to be rare in biologically motivated networks.

We consider a state to be the more *unstable/disordered* the higher the number of its discontent nodes, and we refer to the state as being 1-, 2- up to n-discontent. Intuitively, the system equations tend to drive the system towards the more *stable/ordered* regions of the state space. We note that most of the interesting stable cycles reside in sufficiently ordered space state regions, where a good part of the node values of their members satisfy the equations. Stable cycles including states with a large number of discontent nodes are less relevant which is apparent from the following considerations (compare Figure 3).

If a state of a system with n nodes is k-discontent, it has k successor states, because each one of the k discontent nodes could be updated, and the equations do not change the values of content nodes.

A cycle is stable when all its member states flow within the cycle. A single cycle member having a successor that is not part of the cycle renders the cycle metastable (Figure 3A).

A stable cycle containing a state with even a moderate number of discontent nodes is necessarily a long cycle (state “c” in Figure 3B is 3-discontent); this state has multiple successors and all of them must be members of the cycle as well as all their successors, etc. On the other hand, cycles whose members have very few discontent nodes are often short. We refer to a k-cycle to indicate that its most discontent member state(s) is (are) k-discontent states. A 1-cycle, in which each state member has a single successor, is also referred to as a *simple* cycle (Figure 3A, in the absence of the a → g transition). Otherwise it is a *mixed* cycle and in such cycles “branching” is present (Figure 3B). Simple cycles are necessarily stable. A typical example is: a 1-cycle that consists of 4 states distinguishable by the 4 combinations (0–0, 0–1, 1–0, and 1–1) of the values of two nodes. The remaining k-2 nodes in the network are all content and shared by all the 4 cycle states.

We note that, in principle, there can be cycles that are long despite the fact that all their states have few discontent nodes.

#### ***Stable cycle finding***

The fixed point search described above is performed in states that satisfy all the system equations. It cannot be used to detect a cycle since each member state of a cycle has to be at least 1-discontent. It follows naturally that relaxing the above restriction can be used for cycle finding. Namely, a search in the enlarged subspace that includes all the states up to and including k-discontent states will result in the detection of all j-cycles, where j ≤ k). Unavoidably, the enumeration of more states results in a reduction of the size of resolvable networks.

The value of k can vary from k = 1 (1-cycle search) to n (search for all cycles). The number of selected states grows very fast as k increases. Cycle finding is feasible only when the number of selected states is manageable because of memory restrictions. In addition, execution running times increase fast because the algorithm, in line with the “standard” graph-theoretical approaches, first finds all parent–child state relationships and then searches for stable cycles. If and when one is found, it “creates” its basin of attraction via a bottom-up approach.

The algorithm goes through two main stages:

(i) Determine the appropriate subset of the state space.

a. As in the fixed state case, go through iterations adding a node at a time and keeping at each step all states up to and including k-discontent states. Each state is labeled by its number of discontent nodes (in {0, 1, …, k}). The end result is the set of all these states. The current implementation can readily handle sets with up to tens of millions states. Note that all network fixed states are included and labeled by zero.

a. The predecessors and successors of each selected state are recorded in appropriate data structures. All states that do not have any of their successors in the selected set of states (i.e., they flow “outside”) cannot be assessed and are from now on excluded.

(ii) Search for cycles within the selected set of states:

a. First, identify the basin of attraction of each fixed state. This is done by “walking uphill” the collected information on successor/predecessor relationships, labeling and counting along the way all states belonging to the current basin. Any remaining, non-labeled states belong exclusively to basins of cyclic attractors. In subsequent steps, the search is restricted to these states (apart from the basin size calculations). Absence of non-labeled states clearly implies the absence of cycles in the selected state set; the search is effectively terminated at this point.

a. Starting from state(s) with the lowest k-value, look for directed cycles using again the successor/predecessor data structures, by creating all directed walks in the graph of selected states. If any such cycle is found then it is checked whether any of its member states belong to any of the currently detected basins. If this is the case then the cycle is discarded as unstable. Otherwise, check whether the cycle is “closed”, i.e., whether the children of all its members are also members of the cycle. If this is not the case, the cycle either has members (or successors) belonging to a basin that was not yet found or it is part of a larger mixed cycle. At the current step, such a cycle is simply registered; it will be dealt with at a later stage. If the cycle is found to be closed, then it is stable and constitutes an attractor. All its basin states are counted and any non-previously labeled ones are now labeled. (Note that a state can belong to multiple attractors.) This step is repeated until all registered states have been considered.

a. Detected cycles, whose members do not flow towards identified basins, can still remain. Each one of them may be part of a larger mixed cycle. An iteration process is called which, starting from an appropriately chosen “seed” cycle, keeps enlarging it by adding any new states from cycles with states flowing into it. The iterations continue until this enlarged cycle becomes “closed”. As previously, the corresponding basin is determined and the process continues for any remaining non-labeled states.

The time needed for the exact determination of all successor/predecessor relationships and for the enumeration of the members of a basin grows linearly with the number of selected states. This fact is instrumental in rendering the algorithm fast. Another crucial element is that the size of the search space is reduced each time an attractor basin is determined.

### Benchmarking

For benchmarking purposes we generated programmatically a number of sufficiently complex Boolean networks that have features similar to known, biologically relevant examples. As mentioned above, nodes with a single incoming and a single outgoing edge were excluded. The ratio of edges to nodes was around 2.5 for most of the networks (when not then it is stated so) and no more than 6 incoming edges per node were allowed. We chose to compare *lnet* to *GenYsis* and *GINsim* because these two packages support both true asynchronous updating and guarantee detection of all cycles (for networks up to certain size)*. lnet* and *GenYsis* share a common input format; for *GINsim* a compatible version was generated for all relevant runs.

**Figure 4:**
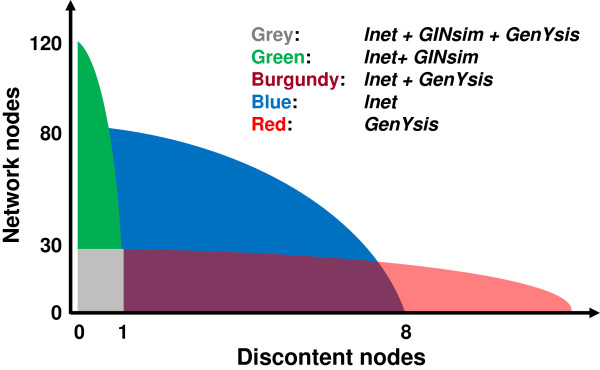
**Schematic comparison of the three algorithms.** Colored regions indicate attractors that can be analyzed using any of the three tools *GenYsis*, *GINsim* and *lnet*. For the region in blue, attractors having at least one discontent node and more than 30 nodes overall, only lnet are applicable.

Only the *lnet* test runs, which involved hundreds of networks, were automated The *GenYsis* runs, as seen below, were limited to smaller-size networks while the *GINsim* ones were difficult to automate. Therefore, we ran just a few of them that still generate sufficiently representative results.

The tests were performed on a Linux computer with a Xeon 2.4 GHz processor and 96 GB of physical memory. The GINsim version was 2.4 and it ran on Java 1.5.

*GenYsis* was found to be superior in detecting all fixed points and all cycles for networks up to approximately 30 nodes. The execution time for the larger ones can reach 1 hour and the memory requirements are minimal. For even larger networks the execution times were higher than our testing time (1 hour) and memory utilization was becoming important. These results are in overall agreement with those of Ferhat et al. [17]. It should be emphasized that *GenYsis* by design detects all existing cycles, also covering cycles with thousands of member states.

*GINsim* detects fixed states for networks up to approximately 120 nodes fast and in a memory-efficient manner as previously reported by Hinkelman [18]. Being multithreaded, *GINsim* took also advantage of our multicore testing environment. The execution time for larger networks was, however, higher than our testing time and sometimes seemed not to terminate at all. Its performance when searching for stable cycles was rather poor. It needs already few minutes to analyze 20-node networks and fails for even moderately larger networks. The reason is that it attempts exhaustive enumeration of all network states. Here it should be noted that *GINsim* can handle multiple-valued networks too.

*lnet* detects fixed states for networks up to 150 nodes, detects all cycles for networks up to 20 or so nodes and detects a subset of the cycles for networks with up to 70 nodes. The limiting factor is available computer memory because the size of the space state regions enumerated by *lnet* grows, inevitably, too large.

For cycle detection, up to 20-node networks, *lnet*’s performance is equivalent to that of *GenYsis*. From 20 to 30 nodes it is still the same provided, however, that the *lnet* search is restricted to k-discontent states with low k (the actual value of k depends on the network size). Otherwise, it can be significantly slower, for similar reasons as *GINsim*. The slowdown can be important in the absence of attractors with very low k (k = 0 or 1) because these are always detected first and have their basin states immediately removed from any downstream consideration. Elucidating further the different performances of the two algorithms, a 30-node network analysis may take several hours in *GenYsis* irrespective of the numbers of fixed states or cycles (no matter how large). On the other hand, while *lnet* may fail to detect a very large cycle, it will find fixed states and small cycles in sub-second time.

Above 30 or so nodes *lnet* is alone in detecting cycles. For increasing numbers of nodes, we have to decrease the allowed number of discontent nodes for any enumerated state because of limited computer memory. This results in fewer detectable cycles. Recall, however, that k-cycles with low k values are likely to be the most relevant ones in Boolean networks representing biological systems.

A schematic representation of the comparison of the three algorithms is shown in Figure 4. The remaining figures focus exclusively on *lnet* results.

**Figure 5:**
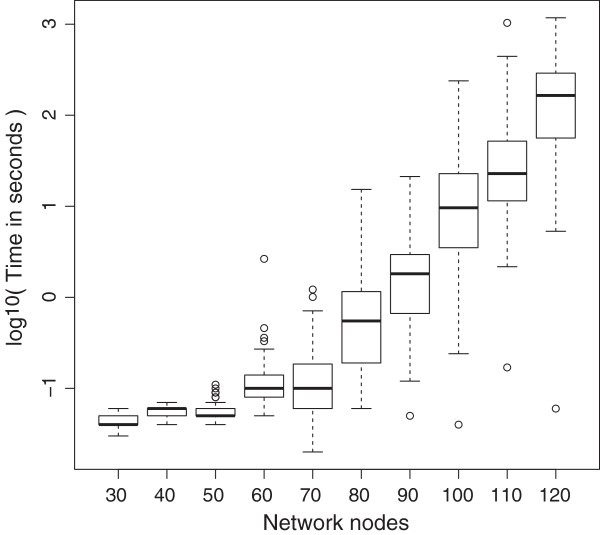
**Dependence of the duration of fixed state searches on the number of network nodes.** Explanation in the main text.

**Figure 6:**
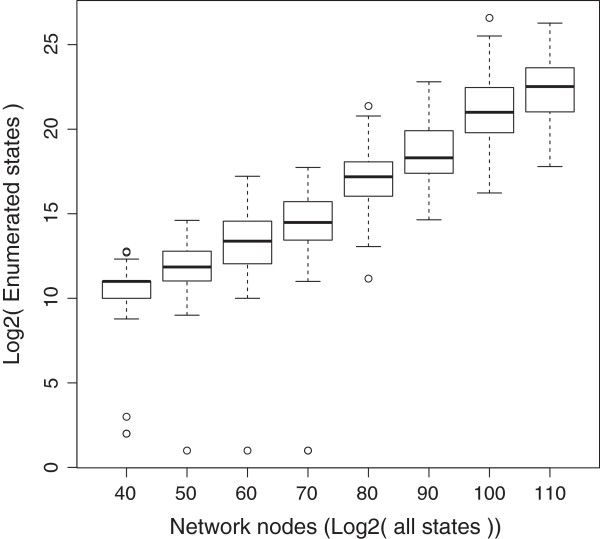
**Enumerated states versus the total number of states.** This log_2_-log_2_ plot clearly demonstrates the significant reduction in the number of analyzed states computed to all possible network states.

**Figure 7:**
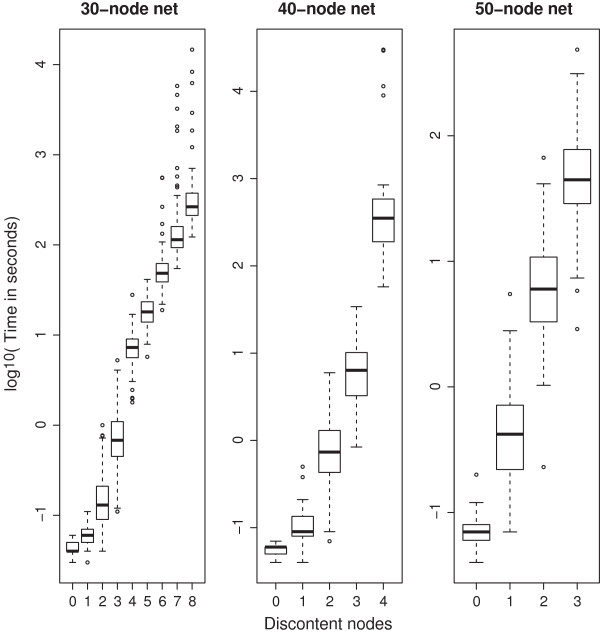
**The number of accessible discontent nodes decreases as the number of nodes grows.** Computation time for analyses of networks of networks of different size, depending on the maximum allowed number of discontent nodes. For larger networks, there is a gradual reduction of the maximum number of discontent nodes the software can handle. This is also mirrored in an increased use of memory (not shown). For example, for 40-node networks cycles with up to 4 discontent nodes were accessible. Such cycles contain tens or hundreds of states.

**Figure 8:**
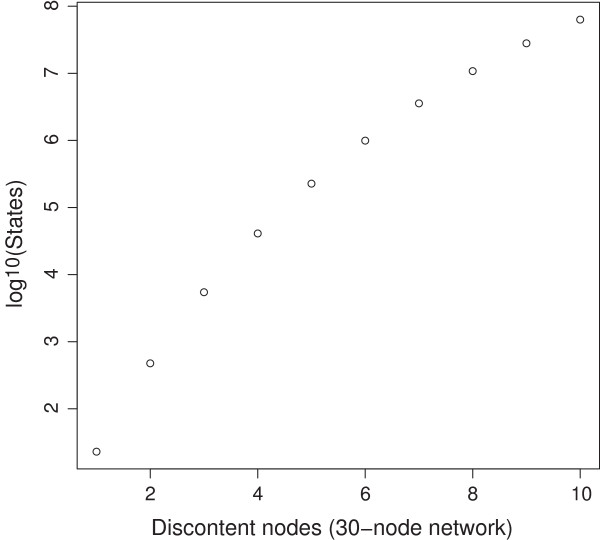
**Even for cycle searches the numbers of enumerated states remain low.** For a 30-node network with approximately 10^9.03^ states, the number of states with no more than 10 discontent nodes still remains a small fraction of the state space.

**Figure 9:**
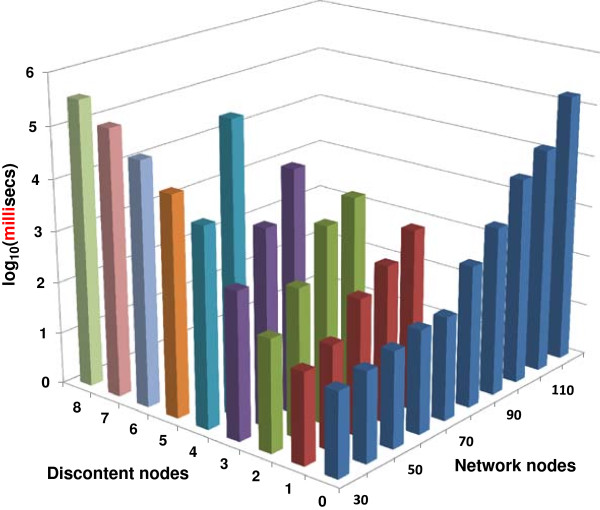
**Overall *lnet *performance. ***lnet* execution time dependence on network size and number of discontent nodes.

**Figure 10:**
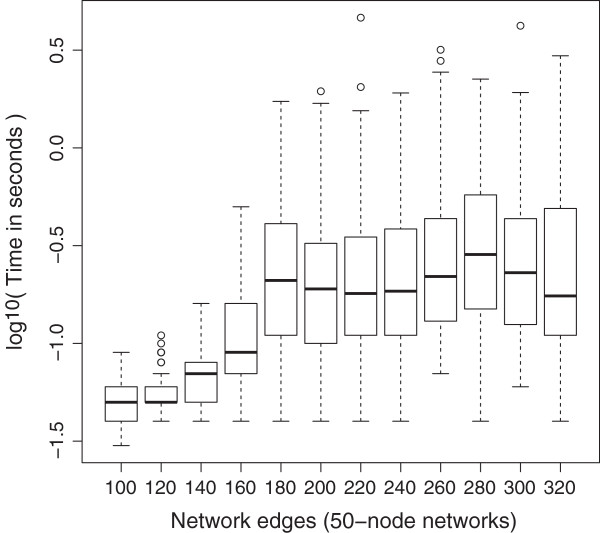
**Effect of the number of edges on fixed state searches in 50-node networks.** As the number of edges increases the median *lnet* execution time increases only slightly but more network instances take substantially longer to analyze (outlying points).

The first series of tests targeted the fixed states of networks having nodes ranging from 30 up to 150. For each one node number analyzed, a set of 100 networks was generated, subject to the rules mentioned earlier. Then each one of these networks was analyzed by *lnet* either searching exclusively for fixed states or for stable cycles as well. The computer execution times were recorded and the results were used to generate the plots shown in Figures 5, 6, 7, 8,9 and 10.

In Figure 5 a semi-log plot of the number of nodes versus the computer execution time in seconds is shown. Data are not shown for the 130, 140 and 150 cases because insufficient memory caused termination for 20%, 68% and 68% of them respectively. (For those networks that were successfully analyzed the time duration trends were in agreement with the trend shown in the plot.)

Next, as has been already explained, the *lnet* algorithm relies on a drastic reduction of the number of enumerated states. This becomes evident in Figure 6, where the enumerated states are plotted versus the total number of system space states (for fixed state searches only).

In Figure 7, the focus is on cycle searches. The 3 plots show computation times for cycle searches in networks of 30, 40 and 50 nodes, respectively. Data from higher number of nodes (up to 70) are not shown but follow similar trends.

Figure 8, also concerning cycle searches, shows counts of system states having equal or less numbers of discontent nodes, for increasing numbers of discontent nodes. For a single 30-node network, states with up to 10 discontent nodes still cover less than 6% of the entire state space. Enumerating this fraction is sufficient to detect existing cycles with up to a few thousand member states.

Figure 9 summarizes all the *lnet* results concerning both fixed states and cycles.

Finally, Figure 10 shows the effect of increasing the number of edges for networks with a fixed number of nodes (50 nodes were chosen).

## Conclusion

We present an algorithm that, based on a build-up approach, greatly reduces the search space for fixed states and fixed cycles in Boolean networks. It allows for the fast and reliable detection of all fixed states in networks of up to 150 nodes, for an edges-to-nodes ratio of up to 3. Highly ordered cycle attractors, which correspond to the biologically most relevant cycles, are detected for networks of approximately half the number of nodes.

Key characteristics of the approach are the exact enumeration of well-defined regions of the network state spaces and the faithful reproduction of the corresponding state transition graphs. The result is the detection of all the attractors residing in these regions. Depending on the overall fraction of the enumerated subspaces, it may be possible to deduce information about the size of each attractor basin.

Execution times of *lnet* are comparable to or faster than other approaches. Its limitation is clearly the available memory. With growing network size, even focusing on small sub-spaces of the entire network state space becomes prohibitively complex at some point. Programming-inspired optimizations will only lead to marginal improvements because the state space of a Boolean network doubles in size each time a single node is added. A promising alternative is to apply first one of the existing network reduction methodologies and then submit the resulting network to *lnet*. Such a scenario should be able to handle networks having (prior to reduction) up to few hundred nodes.

During the preparation of the manuscript we became aware of a publication by SQ Zhang et al. [19], where a fixed state detection algorithm, similar to the one presented here, is described. The authors also provide a conceptual outline of a cycle-finding version of their algorithm based on a simple periodicity check which, however, could detect reliably only simple cycles. We did not test this algorithm because no sufficient information is available. Finally, there is no discussion of the role of the number of discontent nodes in cycle detection.

## Availability and requirements

The *lnet* executable, a file with instructions on how to use it and examples of input files are provided in Additional file 1.

**Operating system(s)**: The source code can be compiled on Windows, Linux and Apple computers.

**Programming language**: ANSI C

**Restrictions of use by non-academic users**: None

## Competing interests

Nikolaos Berntenis and Martin Ebeling are full-time employees of F. Hoffmann–La Roche Ltd.

Both authors declare that they have no competing interests.

## Authors’ contributions

NB wrote the *lnet* code and performed the comparison tests. NB and ME conceived the algorithm and wrote the manuscript. Both authors read and approved the final manuscript.

## Supplementary Material

Additional file 1 **Includes the *lnet *** **executable (LINUX), a help file (text format) with detailed instructions on how to run *lnet *** **and 4 input network files that are used to illustrate the software in the help file.** Click here for file
