# Supplementary material for: Detection of attractors of large Boolean networks via exhaustive enumeration of appropriate subspaces of the state space
Source: BMC Bioinformatics. 2013 Dec 13;14:361. doi: 10.1186/1471-2105-14-361 (PMC3882777; doi:10.1186/1471-2105-14-361)
Supplement: Additional file 1 — Includes the lnet executable (LINUX), a help file (text format) with detailed instructions on how to run lnet and 4 input network files that are used to illustrate the software in the help file. [file 1471-2105-14-361-S1.zip › Instructions.pdf]

lnet – Help file

This document provides essential information on how to use *lnet.* It discusses the input and output as well as the actual command line with its various flags. Furthermore, it contains two sets of examples. The former illustrates how to generate results analogous to those presented in the submitted manuscript, while the latter describes some additional features.

The following input files are used in the examples:

big_network.txt

default_constraints.txt

small_network.txt

modified_constraints.txt

Input

1 **Network file**

Describes the network submitted for analysis (contains nodes and edges) and must be space separated.

For example, for the *mphage* network file (*small_network.txt* in the examples) contains the lines:

Cl -> Cl

Cl -| Cro

Cl -| Cll

Cl -| N

Cro -| Cro

Cro -| Cl

Cro -| N

Cro -| Cll

N -> Cll

Cll -> Cl

2. **Constraints file (**needed only when searching for attractors**)**

Assigns values (0 or 1) to the network logical parameters and is also space-separated.

It should always contain at least (and start with) the following 3 lines:

basal 0

expected 1

top 1

These lines imply that: (i) The basal values of all logical parameters are set to 0, (ii) all parameter values corresponding to all activators being ON and all inhibitors being OFF are set to 1 and (iii) when one inhibitor is ON it overwrites all activators.

These are the default constraints.

This file can contain additional lines, each one specifying the values of specific logical symbols (that may overwrite the generic case stated above).More details can be found at the last example.

Output

All results are given at the standard output.

They include (depending on the command line flags used):

1. The attractors found.
2. The logical parameters (for small enough networks).
3. The states of the network (for small enough networks).

Command line (Linux)

Copy *lnet* to your local directory and execute: *chmod +x lnet*

The actual command line is:

./lnet –vslX[n] network_file constraints_file

Notes:

1. If interested solely in attractor finding then use only the flag **–X[n]**.

   This flag invokes the algorithms described in the submitted publication.

2. The input number **n** is needed only when the flag **–X** is used. It represents the number of allowed discontent nodes when searching for attractors. If equal to 0 (or omitted) the search is performed for fixed states only.

3. The flags **–v** and **–s** cause the output of information like: logical parameters, network equations, node *inDegrees* and *outDegrees*, system state binary vectors and node loops.

To avoid long outputs the flag **–s** should be used only for small networks. The flag **-v** can be also used for large networks but only together with the flag **-X**.

4. The combination **–vl** returns all node loops (for networks of moderate size)
5. It is important monitor the computer **memory (RAM) usage** when searching for attractors. For more details, look at the results in the submitted manuscript, keeping always in mind that they were generated in a computer with 96 Gbytes of RAM.
6. **Technical note 1**: The provided lnet executable supports cycle detection of networks with up to 63 nodes. There is not any such limit for fixed state detection. An additional *lnet* version that theoretically supports cycle detection up to 112 network nodes is also available. However, as our submitted results show, the *lnet* RAM requirements for cycle detection in networks with more than 60 nodes become prohibitive. All submitted results were, therefore, generated by the provided l*net* version.
7. **Technical note 2**: The provided *lnet* version cannot handle nodes with *inDegree* larger than 8. There is not any difficulty at all, however, to double this value to 16. Furthermore, note that the all submitted results were generated with networks with nodes having *inDegree* smaller or equal to 6.

Examples

First example set (attractor detection for all network sizes)

Consider the (randomly generated) network file big_file.txt that has 50 nodes and 125 edges. The constraint file used contains the default constraints.

For fixed state search

1. **Command line**:

./lnet –X big_network.txt constraints_default.txt

Output:

FIXED STATE ID: 16100 Decimal ID: 1055601122038914 Basin states: 1905 Basin fraction: 0.072

FIXED STATE ID: 22752 Decimal ID: 1055601122038946 Basin states: 10459 Basin fraction: 0.395

For each fixed state the Decimal ID represents the corresponding binary state vector, e.g., 1055601122038914 → 111100000000010000010010011110011010101001 0000010

The order of nodes in the binary vector can be found by employing the flag –vX.

For cycle search up to (and including) 3 discontent nodes:

2, **Command line**:

./lnet –X3 big_file.txt constraints_default.txt

Output (in addition to the two fixed states above) is

MIXED CYCLE: Length/NF=15/3   Member states:

8999_15081_12492_22991_20113_22325_4323_10475_7208_11185_14308_5566_14485_5625_15816   Basin states: 3302   Basin fraction: 0.125

The mixed cycle found consists of 15 states and (at least) one of them has 3 discontent nodes.

The 15 numbers shown above are internal *lnet* ids. In order to get the actual node values of each member state, use the flags –vX. They will give the decimal ID corresponding to each internal id (as well as the possible paths inside the mixed cycle). The flag –X4 can also be used to detect the cycle (assuming that sufficient RAM is available). The outcome will be identical if there are no cycles containing states with 4 discontent nodes On the other hand, the –X2 flag cannot detect the cycle, it outputs the two fixed states.

The above example assumes the default constraints. It is possible, of course, to modify the constraints (i.e., the values of the logical parameters). This is shown in the last example.

Second example set (only for small networks)

The *mphage* network will be used, assumed to be stored in the file small_network.txt. There are three examples here.

1. Command line

./lnet –vs small_network.txt

No values are assigned to the network parameters; no attractor search is performed.

Also, note the absence of the constraints file.

Output (the logical structure of the system**):**

BOOLEAN, 4 nodes, 10 interactions, 24 Ks and 16 states (bit-length=4).

Cl 2 levels 3/4 in/out edges Cl:base=-1 Cl:Cll=-1 Cl:Cro=-1

Cl:Cl=-1 Cl:Cro_Cll=-1 Cl:Cl_Cll=-1 Cl:Cl_Cro=-1 Cl:Cl_Cro_Cll=-1

Cro 2 levels 2/4 in/out edges Cro:base=-1 Cro:Cro=-1 Cro:Cl=-1

Cro:Cl_Cro=-1

Cll 2 levels 3/1 in/out edges Cll:base=-1 Cll:N=-1 Cll:Cro=-1

Cll:Cl=-1 Cll:Cro_N=-1 Cll:Cl_N=-1 Cll:Cl_Cro=-1 Cll:Cl_Cro_N=-1

N 2 levels 2/1 in/out edges N:base=-1 N:Cro=-1 N:Cl=-1

N:Cl_Cro=-1

Cl = Cl(1) + Cro(-1) + Cll(1)

Cro = Cl(-1) + Cro(-1)

Cll = Cl(-1) + Cro(-1) + N(1)

N = Cl(-1) + Cro(-1)

#	Cl	Cro	Cll	N	Cl	Cro	Cll	N
0*	0	0	0	0	Cl:Cro	Cro:Cl_Cro	Cll:Cl_Cro	N:Cl_Cro
1*	0	0	0	1	Cl:Cro	Cro:Cl_Cro	Cll:Cl_Cro_N	N:Cl_Cro
2*	0	0	1	0	Cl:Cro_Cll	Cro:Cl_Cro	Cll:Cl_Cro	N:Cl_Cro
3*	0	0	1	1	Cl:Cro_Cll	Cro:Cl_Cro	Cll:Cl_Cro_N	N:Cl_Cro
4*	0	1	0	0	Cl:base	Cro:Cl	Cll:Cl	N:Cl
5*	0	1	0	1	Cl:base	Cro:Cl	Cll:Cl_N	N:Cl

6*	0	1	1	0	Cl:Cll Cro:Cl Cll:Cl N:Cl
7*	0	1	1	1	Cl:Cll Cro:Cl Cll:Cl_N N:Cl
8*	1	0	0	0	Cl:Cl_Cro Cro:Cro Cll:Cro N:Cro
9*	1	0	0	1	Cl:Cl_Cro Cro:Cro Cll:Cro_N N:Cro
10*	1	0	1	0	Cl:Cl_Cro_Cll Cro:Cro Cll:Cro N:Cro
11*	1	0	1	1	Cl:Cl_Cro_Cll Cro:Cro Cll:Cro_N N:Cro
12*	1	1	0	0	Cl:Cl Cro:base Cll:base N:base
13*	1	1	0	1	Cl:Cl Cro:base Cll:N N:base
14*	1	1	1	0	Cl:Cl_Cll Cro:base Cll:base N:base
15*	1	1	1	1	Cl:Cl_Cll Cro:base Cll:N N:base

2. **Command line** (node loop detection)**:**

```
./lnet –lv small_network.txt
```

Output (node loops, in addition to the logical structure of the system)**:**

----

7 loops were found with 4 or less members

#	Length	Sign	Subspace nodes/dim	Functional	Description
0	1	1	2 / 4	0 / 0	Cl --> Cl
1	1	-1	1 / 2	0 / 0	Cro --| Cro
2	2	1	2 / 4	0 / 0	Cl --| Cro --| Cl
3	2	-1	3 / 8	0 / 0	Cl --| Cll --> Cl
4	3	1	2 / 4	0 / 0	Cl --| Cro --| Cll --> Cl
5	3	-1	2 / 4	0 / 0	Cl --| N --> Cll --> Cl
6&	4	1	0 / 1	0 / 0	Cl --| Cro --| N --> Cll --> Cl

Actually, no information on the loop functionality is provided at this level.

Note the ampersand '&'at the last loop. Its presence signals either a sink or a source loop. The case here is rather trivial; all the network nodes are present in a loop, which can be described, of course, as a sink.

3. **Two scenarios** are presented here. The second shows <u>how to modify the constraints</u>

Command line (fixed state detection**):**

./lnet –v small_network.txt constraints_default.txt

.

Output : (the logical structure of the system plus fixed state detection**)**

BOOLEAN, 4 nodes, 10 interactions, 24 Ks and 16 states (bit-length=4).

Cl 2 levels 3/4 in/out edges Cl:base=0 Cl:Cll=0 Cl:Cro=0 Cl:Cl=0 Cl:Cro_Cll=1 Cl:Cl_Cll=0 Cl:Cl_Cro=1 Cl:Cl_Cro_Cll=1

Cro 2 levels 2/4 in/out edges Cro:base=0 Cro:Cro=0 Cro:Cl=0 Cro:Cl_Cro=1

Cll 2 levels 3/1 in/out edges Cll:base=0 Cll:N=0 Cll:Cro=0 Cll:Cl=0 Cll:Cro_N=0 Cll:Cl_N=0 Cll:Cl_Cro=0 Cll:Cl_Cro_N=1

N 2 levels 2/1 in/out edges N:base=0 N:Cro=0 N:Cl=0 N:Cl_Cro=1

Cl = Cl(1) + Cro(-1) + Cll(1)

Cro = Cl(-1) + Cro(-1)

Cll = Cl(-1) + Cro(-1) + N(1)

N = Cl(-1) + Cro(-1)

Number of states=16 possible *fixed=1 free Ks=4

#	Cl	Cro	Cll	N	Cl	Cro	Cll	N
0	0	0	0	0	0	1	0	1
1	0	0	0	1	0	1	1	1
2	0	0	1	0	1	1	0	1
3	0	0	1	1	1	1	1	1
4	0	1	0	0	0	0	0	0
5	0	1	0	1	0	0	0	0
6	0	1	1	0	0	0	0	0
7	0	1	1	1	0	0	0	0
8*	1	0	0	0	1	0	0	0
9	1	0	0	1	1	0	0	0
10	1	0	1	0	1	0	0	0
11	1	0	1	1	1	0	0	0
12	1	1	0	0	0	0	0	0
13	1	1	0	1	0	0	0	0
14	1	1	1	0	0	0	0	0
15	1	1	1	1	0	0	0	0

#	Cl	Cro	Cll	N
8*	1	0	0	0	0

State 8 is the <u>detected fixed state</u>.

Constraint modification scenario:

If the constraints file becomes

basal 0

expected 1

top 1

N:Cro 1

Every added constraint overwrites its default value (the N:Cro default value is 0). The N:Cro parameter is chosen here for illustration purposes.

Command line (note the new constraints file**)**

./lnet –v small_network.txt modified_constraints.txt

Output :

BOOLEAN, 4 nodes, 10 interactions, 24 Ks and 16 states (bit-length=4).

Cl 2 levels 3/4 in/out edges Cl:base=0 Cl:Cll=0 Cl:Cro=0 Cl:Cl=0 Cl:Cro_Cll=1 Cl:Cl_Cll=0 Cl:Cl_Cro=1 Cl:Cl_Cro_Cll=1

Cro 2 levels 2/4 in/out edges Cro:base=0 Cro:Cro=0 Cro:Cl=0 Cro:Cl_Cro=1

Cll 2 levels 3/1 in/out edges Cll:base=0 Cll:N=0 Cll:Cro=0 Cll:Cl=0 Cll:Cro_N=0 Cll:Cl_N=0 Cll:Cl_Cro=0 Cll:Cl_Cro_N=1

N 2 levels 2/1 in/out edges N:base=0 **N:Cro=1** N:Cl=0 N:Cl_Cro=1

Cl = Cl(1) + Cro(-1) + Cll(1)

Cro = Cl(-1) + Cro(-1)

Cll = Cl(-1) + Cro(-1) + N(1)

N = Cl(-1) + Cro(-1)

Number of states=16   possible *fixed=1   free Ks=3

#	Cl	Cro	Cll	N	Cl	Cro	Cll	N
0	0	0	0	0	0	1	0	1
1	0	0	0	1	0	1	1	1
2	0	0	1	0	1	1	0	1
3	0	0	1	1	1	1	1	1
4	0	1	0	0	0	0	0	0
5	0	1	0	1	0	0	0	0
6	0	1	1	0	0	0	0	0
7	0	1	1	1	0	0	0	0
8	1	0	0	0	1	0	0	1
9*	1	0	0	1	1	0	0	1
10	1	0	1	0	1	0	0	1
11	1	0	1	1	1	0	0	1
12	1	1	0	0	0	0	0	0
13	1	1	0	1	0	0	0	0
14	1	1	1	0	0	0	0	0
15	1	1	1	1	0	0	0	0

Cl Cro Cll

9*	1	0	0	1	0

The fixed state is different that the one found in the default case, mirroring the modified value of the **N:Cro**.

There is no limit on the number of constraints that can be modified. In this way, implementing complex combinations of AND, NOT and OR operators becomes possible and straightforward, without modifying the syntax of the network file (data now shown).
